# Remote Zone Extracellular Volume and Left Ventricular Remodeling in Survivors of ST-Elevation Myocardial Infarction

**DOI:** 10.1161/HYPERTENSIONAHA.116.07222

**Published:** 2016-07-13

**Authors:** Jaclyn Carberry, David Carrick, Caroline Haig, Samuli M. Rauhalammi, Nadeem Ahmed, Ify Mordi, Margaret McEntegart, Mark C. Petrie, Hany Eteiba, Stuart Hood, Stuart Watkins, Mitchell Lindsay, Andrew Davie, Ahmed Mahrous, Ian Ford, Naveed Sattar, Paul Welsh, Aleksandra Radjenovic, Keith G. Oldroyd, Colin Berry

**Affiliations:** From the BHF Glasgow Cardiovascular Research Centre (J.C., D.C., S.M.R., N.A., I.M., M.M., M.C.P., H.E., S.H., S.W., M.L., A.D., A.M., N.S., P.W., A.R., K.G.O., C.B.) and Robertson Centre for Biostatistics (C.H., I.F.), University of Glasgow, Glasgow, United Kingdom; and Golden Jubilee National Hospital, Dunbartonshire, United Kingdom (D.C., S.W., C.B.).

**Keywords:** edema, extracellular matrix, magnetic resonance imaging, myocardial infarction, myocardium

## Abstract

The natural history and pathophysiological significance of tissue remodeling in the myocardial remote zone after acute ST-elevation myocardial infarction (STEMI) is incompletely understood. Extracellular volume (ECV) in myocardial regions of interest can now be measured with cardiac magnetic resonance imaging. Patients who sustained an acute STEMI were enrolled in a cohort study (BHF MR-MI [British Heart Foundation Magnetic Resonance Imaging in Acute ST-Segment Elevation Myocardial Infarction study]). Cardiac magnetic resonance was performed at 1.5 Tesla at 2 days and 6 months post STEMI. T1 modified Look-Locker inversion recovery mapping was performed before and 15 minutes after contrast (0.15 mmol/kg gadoterate meglumine) in 140 patients at 2 days post STEMI (mean age: 59 years, 76% male) and in 131 patients at 6 months post STEMI. Remote zone ECV was lower than infarct zone ECV (25.6±2.8% versus 51.4±8.9%; *P*<0.001). In multivariable regression, left ventricular ejection fraction was inversely associated with remote zone ECV (*P*<0.001), and diabetes mellitus was positively associated with remote zone ECV (*P*=0.010). No ST-segment resolution (*P*=0.034) and extent of ischemic area at risk (*P*<0.001) were multivariable associates of the change in remote zone ECV at 6 months (ΔECV). ΔECV was a multivariable associate of the change in left ventricular end-diastolic volume at 6 months (regression coefficient [95% confidence interval]: 1.43 (0.10–2.76); *P*=0.036). ΔECV is implicated in the pathophysiology of left ventricular remodeling post STEMI, but because the effect size is small, ΔECV has limited use as a clinical biomarker of remodeling.

**Clinical Trial Registration—:**

URL: https://www.clinicaltrials.gov. Unique identifier: NCT02072850.

## Abstract 2

Supplemental Digital Content is available in the text.

Early after acute ST-elevation myocardial infarction (STEMI), tissue edema and inflammatory cell recruitment occur as a response to myocyte necrosis and systemic inflammation.^1^ The tissue repair response involves remodeling with collagen deposition in both the infarct and remote (noninfarcted myocardium) zones.^1,2^ On the basis of recent developments with cardiac magnetic resonance (CMR) imaging, it is now possible to estimate the percentage extracellular volume (ECV) in defined regions of interest.^3,4^

The pathophysiological and clinical significance of remote zone ECV in survivors of acute STEMI is incompletely understood. To date, studies of remote zone ECV have included patients with chronic myocardial infarction (MI) as a small subgroup (n<50).^3,4^ Furthermore, these studies are cross-sectional, and the changes that may occur in remote myocardium over time have not been investigated.

Our specific aims were to (1) measure ECV repeatedly in STEMI survivors in a longitudinal cohort study; (2) explore the relationships between remote zone ECV 2 days post STEMI and its absolute change at 6 months from baseline (ΔECV) with clinical characteristics of MI severity; and (3) assess whether remote zone ECV or ΔECV are associated with surrogate left ventricular (LV) outcomes during long-term follow-up.

Our objective was to assess the pathophysiological significance of remote zone ECV and LV remodeling in patients after an acute STEMI. We hypothesized that ECV in the remote myocardial regions of patients with STEMI would be associated with measures of severity of MI and 6-month LV volumes. To test this hypothesis, we investigated ECV repeatedly during longitudinal follow-up of STEMI survivors and assessed the associations for remote zone ECV and ΔECV with patient characteristics and LV outcomes.

## Methods

### Design

We enrolled invasively managed patients with acute STEMI^5,6^ into a CMR cohort study in a regional cardiac center between January 3, 2012, and November 22, 2012. The study was approved by the National Research Ethics Service (Reference 10-S0703-28) and was publically registered (NCT02072850^7^). Healthy volunteers also underwent CMR. All the participants provided written informed consent. The Methods section in the online-only Data Supplement provides a detailed description of the protocol and methods.

### Statistics

For a sample size of 110 subjects, a minimum clinically significant correlation between remote zone ECV at baseline and the within-subject change in LV end-diastolic volume at 6 months from baseline could be detected with 90% power and an α of 0.05.

Continuous variables are described as mean±SD if normally distributed, and median (Q1–Q3) otherwise. Categorical variables are described as n (%). Variables were described overall and by tertiles of remote zone ECV. Patient and angiographic characteristics and CMR findings were compared across ECV tertiles using 1-way ANOVA, Kruskal–Wallis tests, or Fisher tests. Variables were compared between patients and healthy volunteers using *t* tests, Mann–Whitney tests, or Fisher tests. Associations between continuous variables were assessed using Pearson correlation coefficient. ECV was compared between segments using *t* tests. Multivariable linear regression analyses were performed to identify associates of remote zone ECV, ΔECV, and LV outcome. Backward selection was performed, and the remaining variables were included in the multivariable models. Linear regression assumptions were verified using standardized residual plots.

Random effects models were used to compute inter-rater reliability measures (interclass correlation coefficient) for the reliability of remote zone ECV values measured independently by 2 observers in 20 randomly selected patients from the cohort. Root mean square error was calculated. Bland–Altman plots were assessed for interobserver reliability and for agreement between synthetic and conventional ECV measures.

Cox proportional hazards regression was used to explore potential associations between ECV and health outcome. The proportional hazards assumption was verified using log-minus-log plots. For these plots, continuous variables were categorized as above and below the median.

All *P* values were 2 sided. A *P* value >0.05 indicated the absence of a statistically significant effect. The natural log was used in transformations of variables. Analyses were performed using SPSS version 22 for Windows (SPSS, Inc, Chicago, IL).

## Results

**Figure 1.**
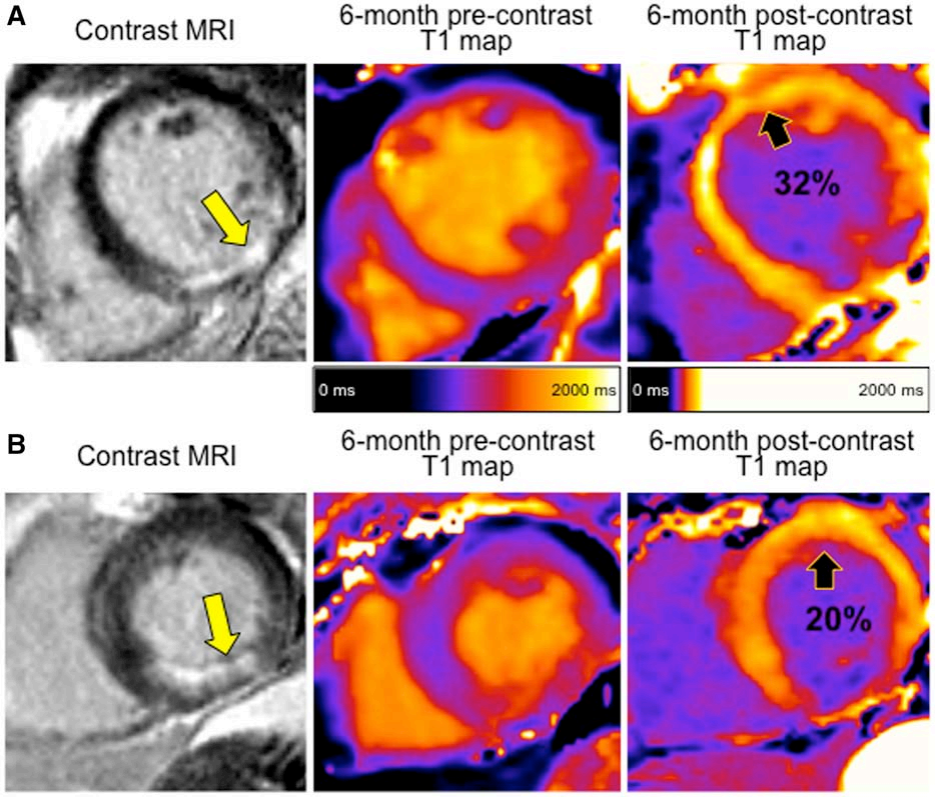
Two patients with similar presentations of acute ST-elevation myocardial infarction (STEMI). Both patients were treated by primary percutaneous coronary intervention and with the same medication. At the end of the procedure, both patients had thrombolysis in myocardial infarction coronary flow grade 3 in the culprit artery. **A**, Increasing remote zone extracellular volume (ECV). Cardiac magnetic resonance (CMR) performed 2 days post STEMI revealed a remote zone ECV of 28%. Remote zone ECV increased by 4% by 6 months to 32%. Left ventricular (LV) end-diastolic volume increased from 116 to 135 mL as measured by the 2-day and 6-month CMR scans. B, Decreasing remote zone ECV: CMR performed 2 days post STEMI revealed a remote zone ECV of 22%. Remote zone ECV decreased by 2% by 6 months to 20%. LV end-diastolic volume decreased from 128 to 102 mL as measured by the 2-day and 6-month CMR scans. MRI indicates magnetic resonance imaging.

One hundred and forty patients with STEMI underwent CMR including pre- and postcontrast T1-mapping at 2.3±1.9 days post revascularization. One hundred and thirty-two patients (94%) had 6-month CMR, of whom 131 (94%) had pre- and postcontrast T1-mapping allowing for the measurement of ECV. Clinical case examples are shown in Figure 1. One thousand six hundred and eighty segments at baseline and 1572 at 6 months were included for analysis. The flow diagram for the study is shown in Figure S1 in the online-only Data Supplement.

### Patient Characteristics

The characteristics of patients with remote zone ECV measurement at baseline (n=140) are described in Table 1. The mean±SD age was 59±11 years, and 76% were male.

**Table 1.**
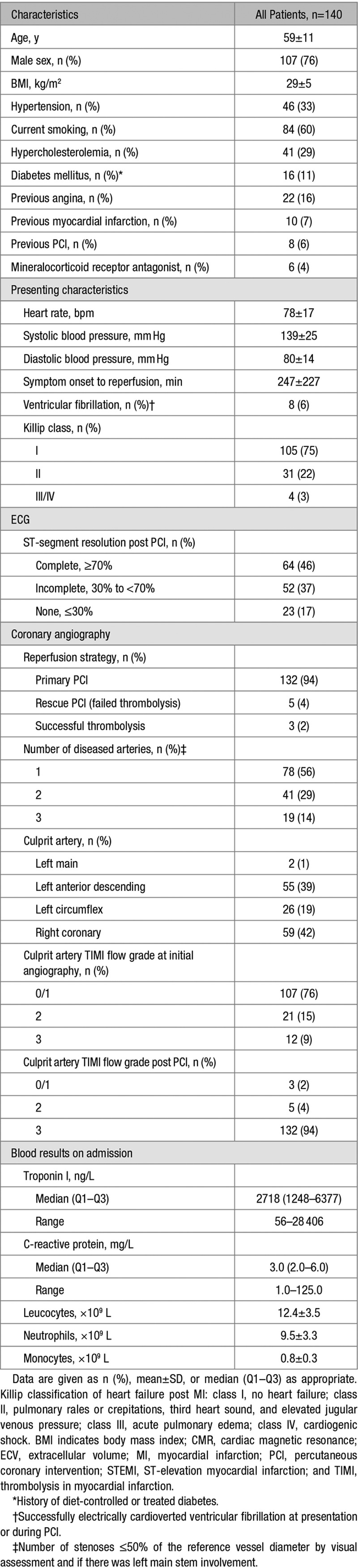
Characteristics of 140 Patients With STEMI With a CMR Measurement of ECV (%) at Baseline

### Myocardial ECV in Patients With STEMI and Healthy Volunteers

Fifteen sex-matched healthy volunteers (age: 60±13 years, 73% male) also underwent CMR assessment of ECV. Remote zone ECV was similar in patients with STEMI (25.6±2.8%) and healthy volunteers (25.4±3.2%; *P*=0.797). In healthy volunteers, ECV was associated with myocardial T2-relaxation time (ms; regression coefficient [95% confidence intervals]: 0.90 [0.38–1.41]; *P*=0.002). Further analysis of healthy volunteer ECV is included in Results in the online-only Data Supplement. The results of interobserver agreement of remote zone ECV measurements are shown in Figure S2.

### Remote Zone ECV and CMR Findings in Patients With Acute STEMI

The tertiles of remote zone ECV were ≤24.2% (n=46), >24.2 to ≤26.4% (n=47), and >26.4% (n=47). The proportion of men decreased with increasing tertile of ECV (43 [94%] versus 35 [75%] versus 29 [62%]; *P*<0.001), and body mass index (BMI, kg/m^2^) reduced with increasing ECV tertile: (30±4 versus 29±5 versus 27±4; *P*=0.018).

Statistically significant CMR findings for patients with baseline ECV (n=140) are summarized in Table 2. The full list of CMR findings are summarized in Table S1. Infarct size was 17±12% of LV mass, and 70 patients (50%) had microvascular obstruction. Remote zone ECV was lower than infarct zone ECV (25.6±2.8% versus 51.4±8.9%; *P*<0.001). The upper tertile of remote zone ECV had values that overlapped with ECV values observed in the infarct zone (Table 2). Remote zone ECV early post MI was positively associated with the extent of myocardial edema (Table 2).

**Table 2.**
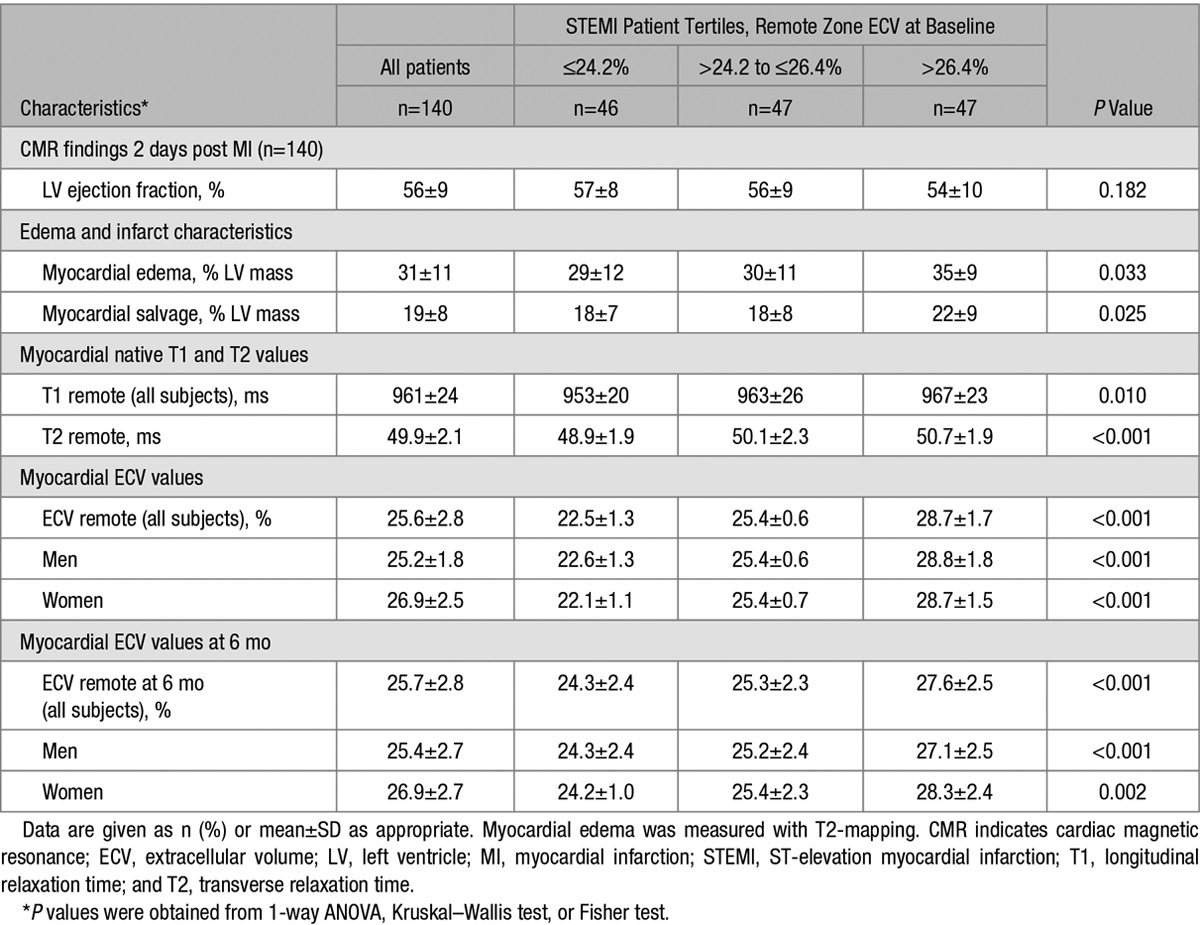
CMR Findings at Baseline (n=140) and at 6 Months (n=131) in Patients With STEMI Grouped by Tertiles of Remote Zone ECV (%) at Baseline

### Multivariable Associations Between Clinical Characteristics and Remote Zone ECV

The multivariable associates of remote zone ECV at baseline are described in Table S2. Male sex (−1.85 [−2.91 to −0.79]; *P*=0001), diabetes mellitus (1.82 [0.43–3.20]; *P*=0.010), BMI (kg/m^2^) (−0.12 [−0.22 to −0.02]; *P*=0.018), and LV ejection fraction at baseline (%) (−0.08 [−0.13 to −0.03]; *P*=0.002) were multivariable associates of remote zone ECV. The extent of myocardial edema (% LV mass) was also a multivariable associate of remote zone ECV (0.09 [0.05–0.14]; *P*<0.001).

### Remote Zone ECV and CMR Findings at 6 Months

**Figure 2.**
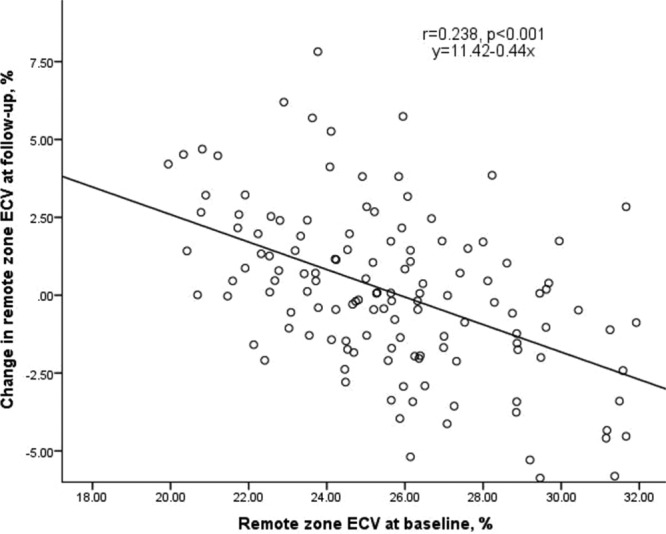
Remote zone extracellular volume (ECV) at baseline versus change in remote zone ECV at follow-up.

The 6-month CMR findings are described in Table 2 and Table S1. For patients with paired observations, remote zone ECV at baseline and follow-up were similar (25.5±2.9% versus 25.7±2.8%; *P*=0.645). The within-subject ΔECV in the remote zone varied markedly (ΔECV 0.1±2.6%). The correlation between remote zone ECV and ΔECV is shown in Figure 2.

At 6 months, LV end-diastolic volume increased on average±SD by 2±25 mL (Table 2) and adverse LV remodeling (an increase in LV end-diastolic volume >20%) developed in 9 patients (7%).

### T1- and T2-Relaxation Times and Remote Zone ECV

Remote zone T2 was marginally higher at follow-up (49.9±2.2 ms versus 50.7±2.4 ms; *P*=0.005; mean change 0.7±3.0 ms). ΔECV in the remote zone was not associated with the change in remote zone T2 (0.07 [−0.01 to 0.14]; *P*=0.068). Remote zone T1 did not change over time.

### Multivariable Associations Between Clinical Characteristics and the Changes in Remote ECV at 6 Months From Baseline

The multivariable associates of ΔECV in the remote zone are shown in Table S3. A 1-U increase in BMI (−0.14 [−0.23 to −0.04]; *P*=0.005), no ST-segment resolution (1.20 [0.09–2.31]; *P*=0.034), and extent of myocardial edema (0.08 [0.04–0.11]; *P*<0.001) were independently associated with ΔECV.

### Remote Zone ECV and LV Remodeling

ΔECV was a multivariable associate of the change in LV end-diastolic volume at 6 months (Table 3); however, this was dependent on size of infarction (% LV mass; Table 3). Baseline remote zone ECV was not associated with LV ejection fraction or volumes at follow-up.

**Table 3.**
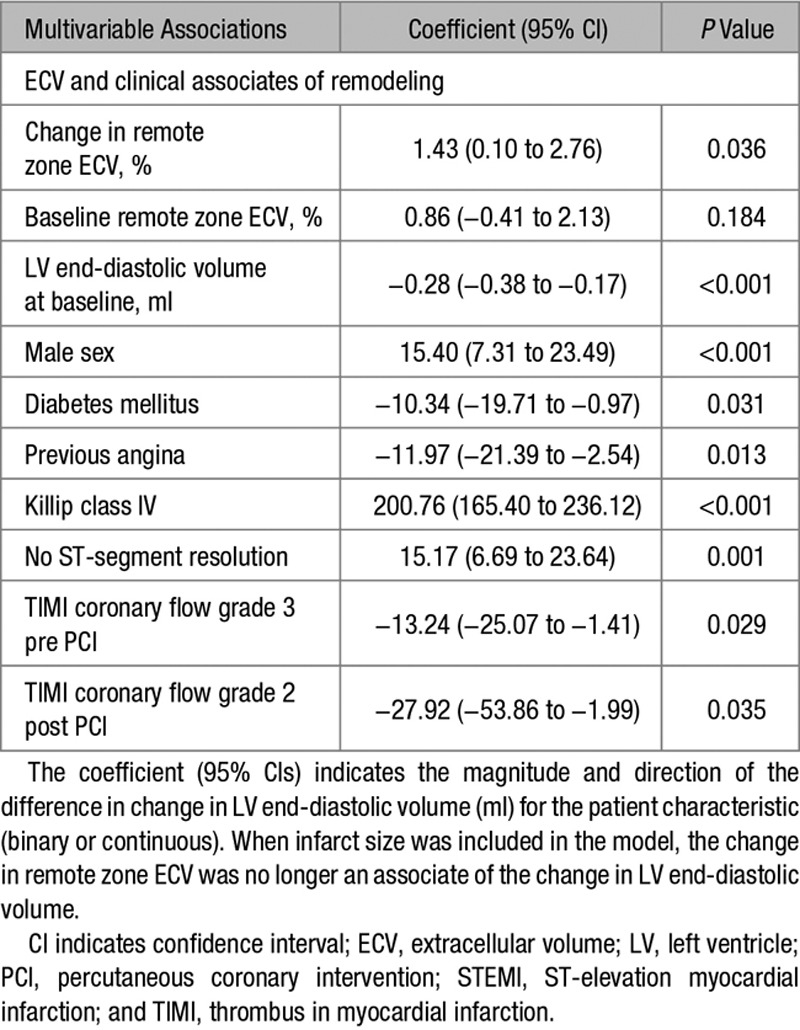
Multivariable Associates of the Change in Left Ventricular End-Diastolic Volume (ml) at 6 Months Post STEMI in 131 Patients

### Remote Zone ECV and NT-proBNP

N-terminal pro-brain natriuretic peptide (NT-proBNP) results were available in 82 subjects. The characteristics of these patients were similar to the whole cohort (data not shown).

Remote zone ECV and NT-proBNP were associated at baseline (0.11 on a log scale [0.03–0.19]; *P*=0.007). This relationship remained after adjustment for LV function and volumes. NT-proBNP at baseline was also associated with ΔECV (0.14 [0.05–0.24]; *P*=0.004) after adjustment for baseline remote zone ECV and independently of LV function and volumes.

### Remote Zone ECV and Health Outcomes

All-cause death or heart failure hospitalization occurred in 7 patients (5%), including 1 death and 6 heart failure episodes. Three patients (2%) experienced an event post discharge. In a preliminary analysis, remote zone ECV was not associated with all-cause death and heart failure hospitalization (n=7).

## Discussion

The main findings are as follows: (1) remote zone ECV 2 days post MI is associated with male sex, BMI, and a history of diabetes mellitus; (2) ΔECV is related to the extent of injury as revealed by myocardial edema; (3) ΔECV was positively associated with changes in LV end-diastolic volume; (4) remote zone ECV and ΔECV were associated with NT-proBNP at baseline. We conclude that in STEMI survivors, an increase in remote zone ECV over time post STEMI is associated with an increase in LV end-diastolic volume, further implicating remote zone interstitial fibrosis in the pathophysiology of adverse LV remodeling.

We recently observed that remote zone tissue characteristics, as revealed by the native myocardial longitudinal relaxation time (T1), were multivariable associates of acute systemic inflammation and subsequent adverse outcomes in the longer term, including LV remodeling and all-cause death or heart failure.^8^ The results from the current study extend these findings in several ways. First, ECV early post MI is inversely associated with the change in ECV over time. Our results indicate that remote zone ECV is likely to remain persistently elevated in patients with a large MI, implying replacement fibrosis and LV remodeling. The absence of an association between remote zone ΔECV and changes in remote zone T2 (ms) over time implies that the persistent elevation in ECV is not explained by edema, indicating that extracellular matrix remodeling and fibrosis is a plausible alternative explanation. Second, the changes over time in remote zone T2 (<1 ms) were biologically insignificant, and remote zone T1 (ms) was unchanged, indicating that the changes in ECV are much more likely because of progressive interstitial fibrosis than interstitial edema. In our previous work, remote zone native T1 was not associated with NT-proBNP,^8^ which is in contrast to the positive association between remote zone ECV and NT-proBNP. These results may be explained by the fact that ECV is a more specific biomarker of the extracellular space, whereas native T1 reflects both intracellular and extracellular compartments. Finally, for the first time, we have reported changes in remote zone ECV over time post MI and the associations with clinical characteristics and LV remodeling. In agreement with previous observations,^8^ the absence of an association between remote zone ECV and the number of stenosed arteries suggest that ischemia is not a factor in remote zone remodeling. In linear regression analyses, λ had similar associations as ECV (data not shown), implying these variables are closely associated.

In this study, remote zone ECV exhibited a sex variation. This is in keeping with studies of remote zone ECV in patients with cardiovascular disease^3,4^ and healthy subjects.^9,10^ Sado et al^10^ also found height to be related to ECV, which agrees with our finding of the link between BMI and ECV. In contrast to findings by Ugander et al,^3^ we observed that remote zone ECV overlapped with the lower limits of infarct zone ECV. The possible explanations for this result include (1) large sample of an unselected real-world STEMI population in our study compared with a smaller sample (n=36) of patients studied by Ugander et al; (2) time between MI and CMR, which was not specified by Ugander et al and may have resulted in higher remote zone ECV in our study because of global edema and inflammatory cell influx, although the overlap persisted at 6-month follow-up.

Our findings are evidence of associations between tissue characteristics in the myocardial remote zone and LV remodeling post STEMI.^3,4,8^ Although the effect size of the changes in ECV limits clinical use, the result is nonetheless important from the perspective of the pathophysiology and mechanisms of LV remodeling. On the contrary, we also observed that the size of infarction was a similar predictor of LV remodeling than remote zone ECV. Whether drugs that inhibit LV remodeling, for example, mineralocorticoid receptor antagonists, might have differential effects in patients according to their baseline ECV status (within the infarct and/or remote zones) merits further research. Wong et al^11^ observed that patients treated with renin-aldosterone-angiotensin system blockade had lower ECV.

### Limitations

Postcontrast T1 maps were not acquired in all patients because of time constraints in our busy clinical service. CMR was performed at a single time point early post MI; therefore, we lack results on the temporal evolution of ECV post reperfusion. Angiotensin-converting enzyme inhibitors were prescribed in almost all patients with STEMI in our cohort (98%), and mineralocorticoid receptor antagonists were prescribed in the minority (4%); therefore, it was not possible to assess for associations between these therapies and ECV post MI. The relationship between remote zone ECV and health outcomes in STEMI survivors merits further study in a larger population. We have undertaken the largest longitudinal study of remote zone ECV in patients after an acute STEMI. Nonetheless, the results do not provide evidence of causality.

### Conclusions

In STEMI survivors, remote zone ECV is associated with sex, BMI, and a history of diabetes mellitus. Remote zone ECV at baseline and ΔECV are associated with the severity of MI including NT-proBNP. Only ΔECV was associated with LV remodeling, as revealed by CMR. ΔECV is implicated in the pathophysiology of LV remodeling post STEMI, but because the effect size is small, ΔECV has limited use as a clinical biomarker of remodeling.

### Perspectives

Our findings provide further evidence of the pathophysiological importance of remote zone tissue characteristics and LV remodeling in survivors of acute STEMI. Future research is warranted into the association between remote zone ECV and longer-term health outcomes. Remote zone ECV is implicated in the pathophysiology of LV remodeling.

## Acknowledgments

We thank the patients and the staff in the Cardiology and Radiology Departments. We thank Peter Weale and Patrick Revell (Siemens Healthcare, UK).

## Sources of Funding

This study was supported by a Project Grant from the British Heart Foundation (BHF; PG/11/2/28474) and the Chief Scientist Office. C. Berry was supported by a Senior Fellowship from the Scottish Funding Council. P. Welsh is supported by BHF Fellowship FS/12/62/29889. This project was also supported by a research agreement with Siemens Healthcare.

## Disclosures

None.

## Supplementary Material

## Novelty and Significance

### What Is New?

- Extracellular volume (ECV) measured by cardiac magnetic resonance imaging has been measured in regions of interest repeatedly over time (2 days and 6 months) in a reasonably large cohort of patients after acute ST-elevation myocardial infarction (MI).
- ECV in the remote zone is associated with patient characteristics and the severity of MI.
- The change in remote zone ECV is associated with left ventricular remodeling.
- Remote zone ECV and the change in remote zone ECV are associated with NT-proBNP, a biochemical marker of MI severity.

### What Is Relevant?

- The change in remote zone ECV is implicated in the pathophysiology of LV remodeling post ST-elevation MI, but because the effect size is small, it has limited use as a clinical biomarker.

### Summary

- Remote zone ECV and the change in remote zone ECV over time are linked with clinical markers of MI severity as evidenced by reperfusion injury and myocardial edema. The change in remote zone ECV is linked with left ventricular remodeling during longer-term follow-up.
