# Supplementary material for: Remote Zone Extracellular Volume and Left Ventricular Remodeling in Survivors of ST-Elevation Myocardial Infarction
Source: Hypertension. 2016 Jul 13;68(2):385–91. doi: 10.1161/HYPERTENSIONAHA.116.07222 (PMC4956675; doi:10.1161/HYPERTENSIONAHA.116.07222)
Supplement: Supplementary file 1 [file hyp-68-385-s001.docx]

**Title:** REMOTE ZONE EXTRACELLULAR VOLUME AND LEFT VENTRICULAR REMODELING IN SURVIVORS OF ST-ELEVATION MYOCARDIAL INFARCTION.

**Short Title:** Remote zone extracellular volume post-STEMI

**Authors**: *Miss Jaclyn Carberry BScMedSci,^1^ *Dr David Carrick MRCP,^1,2^ Dr Caroline Haig PhD,^3^ Mr Samuli Rauhalammi MSc,^1^ Dr Nadeem Ahmed MBChB,^1^ Dr Ify Mordi MRCP,^1^ Dr Margaret McEntegart MRCP,^1^ Dr Mark C. Petrie MRCP,^1^ Dr Hany Eteiba FRCP,^1^ Dr Stuart Hood FRCP,^1^ Dr Stuart Watkins FRCP,^1,2^ Dr Mitchell Lindsay FRCP,^1^ Dr Andrew Davie,^1^ Dr Ahmed Mahrous MD,^1^ Professor Ian Ford PhD,^3^ Professor Naveed Sattar FRCP,^1^ Dr Paul Welsh PhD,^1^ Dr Aleksandra Radjenovic PhD,^1^ Professor Keith G. Oldroyd FRCP,^1^ Professor Colin Berry FRCP.^1,2^ *These authors contributed equally.

**Institutions:** ^1^BHF Glasgow Cardiovascular Research Center, University of Glasgow; ^2^Golden Jubilee National Hospital; ^3^Robertson Centre for Biostatistics, University of Glasgow.

**Correspondence:** Professor Colin Berry, BHF Glasgow Cardiovascular Research Center, 126 University Place, University of Glasgow, Glasgow, G12 8TA, UK. Telephone: +44 (0) 141 330 1671 or +44 (0) 141 951 5000. Fax: +44 (0) 141 330 6794. Email: [colin.berry@glasgow.ac.uk](mailto:colin.berry@glasgow.ac.uk)

**Word count: total =** 4393, (including references, figures and legends), 213 for abstract, 2 figures.

**Table of contents**

Supplemental Methods 2

Supplemental Results 7

References 9

Supplemental Tables 12

Supplemental Figures 19

Supplemental Methods

## Setting and study populations

### ST-elevation myocardial infarction patients

Screening, enrolment, and data collection were prospectively performed by cardiologists in the cardiac catheterization laboratories of the Golden Jubilee National Hospital, Glasgow, United Kingdom. This hospital is a regional referral center for primary and rescue percutaneous coronary intervention (PCI). The hospital provides clinical services for a population of 2.2 million. A screening log was recorded, including patients who did not participate in the cohort study.

### Healthy volunteers

The purpose of including healthy volunteers was to collect normative reference data for myocardial extracellular volume (ECV) in individuals without prior cardiovascular disease or therapy and who were reasonably representative of the population of individuals from whom the ST-elevation myocardial infarction (STEMI) patients were drawn. Second, the reference ECV values were required to be measured on the same CMR scanner and with the same protocol that was used for the STEMI patients including during the same time-period.

Healthy volunteers were invited to participate by placing adverts in public buildings (e.g. hospital, University) and through personal contacts of the researchers. Matching and selection of the healthy volunteers was done by the researchers in order to reflect the age and gender distribution of the STEMI patients. The healthy volunteers were resident in the same catchment area as the STEMI population. Age- and gender-matched healthy volunteers who had a normal electrocardiogram (ECG) and no prior history of cardiovascular disease or therapy underwent CMR during the same time period. The absence of late gadolinium enhancement (myocardial fibrosis or scar) was determined qualitatively by visual assessment, and the absence of late gadolinium enhancement was a requirement for inclusion of the volunteer in this analysis.

The rationale for including healthy volunteers in this study is as follows. First, ECV values may vary between CMR scanners and so a local reference range for ECV is recommended in CMR guidelines [1, 2]. Second, ECV may vary spatially in the heart and therefore, since the focus of our study was to assess ECV in myocardium remote from the infarct zone, we aimed to collect ECV values in different segments of the heart in order to compare the remote zone ECV values from STEMI patients with reference spatially matched remote zone ECV values in age- and gender-matched healthy volunteers. Myocardial ECV values were regionally segmented in regions-of-interest and summarized according to the American Heart Association (AHA) model [3].

## Coronary angiogram acquisition and analyses

Coronary angiograms were acquired during usual care with cardiac catheter laboratory X-ray (Innova®) and IT equipment (Centricity®) made by GE Healthcare (Little Chalfont, UK).

## Percutaneous coronary intervention

Consecutive admissions with acute STEMI referred for emergency percutaneous coronary intervention (PCI) were screened for the inclusion and exclusion criteria. During ambulance transfer to the hospital, the patients received 300 mg of aspirin, 600 mg of clopidogrel and 5000 IU of unfractionated heparin [4, 5]. The initial primary PCI procedure was performed using radial artery access. A conventional approach to primary PCI was adopted in line with usual care in our hospital [4, 5]. Conventional bare metal and drug eluting stents were used in line with guideline recommendations and clinical judgment. The standard transcatheter approach for reperfusion involves minimal intervention with aspiration thrombectomy only or minimal balloon angioplasty (e.g. a compliant balloon sized according to the reference vessel diameter and inflated at 4-6 atmospheres 1-2 times). During PCI, glycoprotein IIbIIIa inhibitor therapy was initiated with high dose tirofiban (25 μg/kg/bolus) followed by an intravenous infusion of 0.15 μg/kg/min for 12 hours, according to clinical judgment and indications for bail-out therapy [4, 5]. No reflow was treated according to contemporary standards of care with intra-coronary nitrate (i.e. 200 μg) and adenosine (i.e. 30 – 60 μg) [4, 5], as clinically appropriate. In patients with multivessel coronary disease, multivessel PCI was not recommended, in line with clinical guidelines [4, 5]. The subsequent management of these patients was symptom-guided.

## Angiographic analysis

The coronary anatomy and disease characteristics of study participants were described based on the clinical reports of the attending cardiologist.

## Outcome definitions

Coronary blood flow can be described based on the visual assessment of coronary blood flow revealed by contrast injection into the coronary arteries [4, 5]. Thrombus in Myocardial Infarction (TIMI) Coronary Flow Grade 0 is no flow, 1 is minimal flow past obstruction, 2 is slow (but complete) filling and slow clearance, and 3 is normal flow and clearance.

## CMR acquisition

CMR was performed on a Siemens MAGNETOM Avanto (Erlangen, Germany) 1.5-Tesla scanner with a 12-element phased array cardiac surface coil.

Myocardial native longitudinal relaxation time (T1) reflects tissue water content and cellularity [2]. T1-mapping was performed pre- and 15 minutes post-gadolinium contrast administration. T1 maps were acquired in 3 short-axial slices (basal, mid and apical), using a modified look-locker inversion-recovery (MOLLI) investigational prototype sequence (Work-in-Progress (WIP) method 448, Siemens Healthcare) [6-8] that incorporates an automatic registration algorithm based on a previously described approach [9]. The MOLLI T1 cardiac-gated acquisition involved three inversion-recovery prepared look locker experiments combined within one protocol (3 (3) 3 (3) 5) [7]. The CMR parameters were: bandwidth ~1090 Hz/pixel; flip angle 35°; echo time (TE) 1.1 ms; T1 of first experiment 100 ms; TI increment 80 ms; matrix 192 x 124 pixels; spatial resolution 2.2 x 1.8 x 8.0 mm; slice thickness 8 mm; scan time 17 heartbeats .

Myocardial T2 relaxation time directly reflects tissue water content and mobility [10,11]. T2-mapping (WIP method 447, Siemens Healthcare) was acquired in contiguous short axis slices covering the whole ventricle, using an investigational prototype T2-prepared (T2P) TrueFisp sequence [10, 11]. The CMR parameters were: bandwidth ~947 Hz/pixel; flip angle 70°; T2 preparations: 0 ms, 24 ms, and 55 ms respectively; matrix 160 x 105 pixels; spatial resolution 2.6 x 2.1 x 8.0 mm; slice thickness 8 mm.

Late gadolinium enhancement images covering the entire LV were acquired 10-15 minutes after intravenous injection of 0.15 mmol/kg of gadoterate meglumine (Gd^2+^-DOTA, Dotarem, Guebert S.A.) using segmented phase-sensitive inversion recovery (PSIR) turbo fast low-angle shot [12]. Microvascular obstruction (MVO) was defined as a dark zone on early delayed enhancement imaging 1, 3, 5 and 7 minutes post-contrast injection and within an area of late gadolinium enhancement at 10-15 minutes. Typical imaging parameters were: bandwidth ~130 Hz/pixel, flip angle 25°, TE 3.36 ms, matrix 192 x 256 pixels, , echo spacing 8.7ms and trigger pulse 2. The voxel size was 1.8 x 1.3 x 8 mm^3^. Inversion times were individually adjusted to optimize nulling of apparently normal myocardium (typical values, 200 to 300 ms).

Healthy volunteers underwent the same imaging protocol, with the exception that those <45 years did not receive gadolinium.

## MR image analyses

The images were analyzed on a Siemens work-station by observers with at least 3 years CMR experience (N.A., D.C., I.M, S.R.). All of the images were reviewed by experienced CMR cardiologists (C.B., N.T.). Left ventricular (LV) dimensions, volumes and ejection fraction were quantified using computer assisted planimetry (syngo MR®, Siemens Healthcare, Erlangen, Germany). All scan acquisitions were spatially co-registered.

### ECV measurement

LV contours were delineated with computer-assisted planimetry on the best spatially matched raw T1 image and copied onto color-coded spatially co-registered maps. Care was taken to have adequate margins of separation from tissue interfaces, such as myocardium and blood, to prevent partial volume averaging [1, 2, 13]. Regions-of-interest (ROIs) were drawn in 1) remote myocardium, placed 180^o^ from infarct zone; 2) infarct zone, including the entire area of injury; 3) LV blood pool. ROIs were copied between the pre- and post-contrast T1 maps with manual correction to maintain margins of separation from tissue interfaces. ECV was calculated as a ratio of corresponding T1 values measured pre- and post- contrast in each of the ROIs. No registration between T1 maps was therefore required for accurate calculation of ECV values. ECV was calculated using Eq. (1), where Lambda ($\lambda$)=ΔR1_myocardium_/ΔR1_blood,_ ΔR1=R1_post-contrast_-R1_pre-contrast_, R1=1/T1 [14, 15]. Hematocrit (HCT) was measured at the time of scanning.

$ECV=\left( 1-HCT \right) \times\lambda$ (1)

ECV analysis in the healthy volunteer cohort was calculated for 6 equal segments (anterior, anterolateral, inferolateral, inferior, inferoseptal, anteroseptal) of the mid-ventricular T1 maps according to the 17-segment model of the American Heart Association (AHA) [3]. Per-segment values were averaged to give a global ECV for each case. To expand the healthy volunteer cohort, synthetic HCT was calculated using the equation HCT = 0.88-(T1_blood_/3240) [16].

### Infarct definition and size

The presence of acute infarction was established based on abnormalities in cine wall motion, rest first-pass myocardial perfusion, and delayed-enhancement imaging. In addition, supporting changes on the ECG and coronary angiogram were also required. Acute infarction was considered present only if late gadolinium enhancement was confirmed on both the axial and long axis acquisitions. The territory of infarction was delineated using a signal intensity threshold of >5 standard deviations (SD) above a remote reference region and expressed as a percentage of total LV mass [13]. Infarct regions with evidence of MVO were included within the infarct area and the area of MVO was assessed separately and also expressed as a percentage of total LV mass.

### Microvascular obstruction

MVO was defined as a dark zone on early gadolinium enhancement imaging 1, 3, 5 and 7 minutes post-contrast injection that remained present within an area of late gadolinium enhancement at 10-15 minutes. The late time-point was used to delineate MVO [1]. Identification of MVO was performed independently by I.M. and N.A. MVO area was assessed separately and expressed as a percentage of LV mass.

### Myocardial edema

The extent of myocardial edema was defined as LV myocardium with pixel values (T1/T2) >2 SD from remote myocardium [17-18]. In order to assess myocardial edema the epicardial and endocardial contours on the last corresponding T2-weighted raw image with an echo time of 55 ms were planimetered [19]. Contours were then copied to the map and corrected when necessary by consulting the SSFP cine images.

Myocardial salvage

Myocardial salvage was calculated by subtraction of percent infarct size from percent myocardial edema (a retrospective estimate of the initial ischemic area-at-risk) [20-22]. The myocardial salvage index was calculated by dividing the myocardial salvage area by the initial extent of edema (% LV mass).

### Adverse remodeling

Adverse remodeling was defined as an increase in LV end-diastolic volume ≥ 20% at 6 months from baseline [23].

## Reference ranges

Reference ranges used in the laboratory were 105 – 215 g for LV mass in men, 70 – 170 g for LV mass in women, 77 – 195 ml for LV end-diastolic volume in men, 52 – 141 ml for LV end-diastolic volume in women, 19 – 72 ml for LV end-systolic volume in men and 13 – 51 ml for LV end-systolic volume in women.

## Electrocardiogram

A 12 lead ECG was obtained before coronary reperfusion and 60 minutes afterwards with Mac-Lab® technology (GE Healthcare) in the catheter laboratory and a MAC 5500 HD recorder (GE Healthcare) in the Coronary Care Unit. The ECGs were acquired by trained cardiology staff. The ECGs were de-identified and transferred to the local ECG management system. The ECGs were then analyzed by the University of Glasgow ECG Core Laboratory which is certified to ISO 9001: 2008 standards as a UKAS Accredited Organization.

The extent of ST-segment resolution on the ECG assessed 60 minutes after reperfusion compared to the baseline ECG before reperfusion [4] was expressed as complete (≥70%), incomplete (30% to < 70%) or none (≤30%).

## Biochemical assessment of infarct size

Troponin T was measured (Elecsys Troponin T, Roche) as a biochemical measure of infarct size. The high sensitive assay reaches a level of detection of 5 pg/ml and achieves less than 10% variation at 14 pg/ml corresponding to the 99th percentile of a reference population. A blood sample was routinely obtained 12 – 24 hours after hospital admission, and again between 0700 – 0900 hours during the first two days of the index hospitalization.

## Biochemical measurement of LV remodeling

Serial systemic blood sample were obtained immediately after reperfusion in the cardiac catheterization laboratory, and subsequently between 0600 - 0700 hrs each day during the initial in-patient stay in the Coronary Care Unit.

NT-proBNP, a biochemical measure of LV wall stress, was measured in a research laboratory using an electrochemiluminescence method (e411, Roche) and the manufacturers calibrators and quality control material. The limit of detection is 5 pg/ml. Long-term coefficient of variations of low and high controls are typically <5%, and were all within the manufacturers range.

## Research Management

The study was conducted in line with Guidelines for Good Clinical Practice (GCP) in Clinical Trials [24].

Research management included a Management Group, and an independent Clinical Trials Unit. Day to day study activity was coordinated by the Management Group who was responsible to the Sponsor which was responsible for overall governance and that the trial was conducted according to GCP standards.

Supplemental Results

The flow diagram for the study is shown in Supplemental Fig. S1.

## Remote zone ECV inter-observer reliability

Remote ECV in a subgroup of 20 randomly chosen patients was independently measured by two observers. The ICC for reliability of remote zone ECV was 0.92 (95% confidence interval (CI): 0.79, 0.97); p<0.001). The mean absolute difference between measures was 0.48%, and the root-mean-square error was 1.01. Bland-Altman plots showed no evidence of bias (Supplemental Fig. S2). The coefficient of variation for values of remote zone ECV at baseline in STEMI patients (n=140) was 13.36.

## Remote zone ECV in healthy volunteers

Twenty-eight healthy volunteers (mean±SD 60±11 years, 16 (57%) male) with no history of cardiovascular disease or treatment had CMR scans including post-contrast T1-mapping with MOLLI. Volunteers were scanned using the same 1.5 Tesla MRI scanner (Siemens AVANTO) as the STEMI patients. Twenty patients had available HCT measures on the day of scanning. For these patients, synthetic ECV and conventional ECV were highly correlated (R^2^=0.82, p<0.001). The correlation was similar for synthetic and conventional remote ECV in STEMI patients (R^2^=0.68, p<0.001). SD of differences for healthy volunteers was 1.3%. The Bland-Altman plot showed no evidence of bias (Supplemental Fig. S3). Regression coefficients between synthetic and conventional ECV measurements were similar. We proceeded to calculate synthetic HCT and ECV for the remaining healthy volunteers, giving a total of n=28 healthy volunteers.

At the mid ventricular level, remote zone synthetic ECV was similar in STEMI patients and healthy volunteers for a sex-matched grouped (n=21, age 58±12 years, 76% male) (24.2±2.7% vs. 24.1±2.6%; p=0.959). In all healthy volunteers (n=28), remote zone ECV was higher in females than in males (25.6±2.4% vs. 23.0±1.7%; p=0.002). Overall, remote zone ECV was negatively associated with age (-0.09 (-0.16, -0.01); p=0.027), however the association was sex-dependent (women: -0.16 (-0.27, -0.04); p=0.015; men: -0.03 (-0.11, 0.06); p=0.498). There was a trend to interaction between age and sex when assessed using linear regression (p=0.052). ECV was higher in septal segments in females (anteroseptal: 25.8±3.0% vs. 22.9±1.9%; p=0.004; inferoseptal: 25.9±2.9% vs. 22.6±1.8%; p=0.001), whereas no differences were observed for other segments (anterior: 24.1±3.4% vs. 22.0±1.9%; p=0.077; anterolateral: 23.9±3.7% vs. 23.0±1.8%; p=0.457; inferolateral: 25.7±3.6% vs. 24.5±2.2%; p=0.309; inferior: 25.3±3.8% vs. 23.8±2.6%; p=0.283).

The coefficients of variation for remote zone ECV in the mid-ventricular level with regions-of-interest within myocardial regions were: anterior CoV = 12.54; anterolateral CoV = 12.09; anteroseptal CoV = 11.53; inferior CoV = 13.26; inferolateral CoV = 11.55; inferoseptal CoV = 11.83.

## Univariable associates with the change in LV end-diastolic volume at 6 months in all patients

Characteristics also included in the model include age (p=0.437), BMI (p=0.437), cigarette smoking (p=0.394), history of hypertension (p=0.846), hypercholesterolemia (0.958), previous PCI (p=0.150), previous MI (p=0.057), heart rate (p=0.183), systolic blood pressure at initial angiography (p=0.848), symptoms to reperfusion time (p=0.210), sustained ventricular arrhythmia (p=0.956), TIMI coronary flow grade 2 pre-PCI vs. TIMI coronary flow grade 0/1 pre-PCI (reference category) (p=0.465), rescue PCI vs. primary PCI (reference category) (p=0.525), incomplete ST-segment resolution vs. complete ST-segment resolution (reference category) (p=0.474), TIMI coronary flow grade 3 post-PCI vs. TIMI coronary flow grade 0/1 post-PCI (reference category) (p=0.191), Killip class II vs. Killip class I (reference category) (p=0.239), Killip class III vs. Killip class I (reference category) (p=0.110).

The multivariable predictors of the change in LV end-diastolic in all patients are described in Table 3 of the main paper.

## Remote zone ECV and health outcomes in the longer term

Health outcome data was available in 140 (100%) patients. The median duration of follow-up was 724 days (minimum-maximum post-discharge censor duration 598- 923 days). Twelve (9%) patients experienced a major adverse cardiac event (MACE), including 6 recurrent MI hospitalizations and 6 heart failure episodes (hospitalization with Killip Class 3 or 4 heart failure or defibrillator implantation). Seven (5%) patients experienced a MACE post-discharge. Remote zone ECV was not associated with MACE (n=12). All-cause death or heart failure hospitalization occurred in 7 (5%) patients, including 1 death and 6 heart failure episodes. Three (2%) patients experienced an event post-discharge. Remote zone ECV was not associated with all cause death and heart failure hospitalization (n=7) (hazard ratio (95% confidence interval) 1.08 (0.73, 1.59)).

References

1. Kramer CM, Barkhausen J, Flamm SD, Kim RJ, Nagel E; Society for Cardiovascular Magnetic Resonance Board of Trustees Task Force on Standardized Protocols. Standardized cardiovascular magnetic resonance (CMR) protocols 2013 update. *J Cardiovasc Magn Reson*. 2013;15:91.
2. Moon JC, Messroghli DR, Kellman P, Piechnik SK, Robson MD, Ugander M, Gatehouse PD, Arai AE, Friedrich MG, Neubauer S, Schulz-Menger J, Schelbert EB; Society for Cardiovascular Magnetic Resonance Imaging, Cardiovascular Magnetic Resonance Working Group of the European Society of Cardiology. Myocardial T1 mapping and extracellular volume quantification: a Society for Cardiovascular Magnetic Resonance (SCMR) and CMR Working Group of the European Society of Cardiology consensus statement. *J Cardiovasc Magn Reson*. 2013;15:92.
3. Cerqueira MD, Weissman NJ, Dilsizian V, Jacobs AK, Kaul S, Laskey WK, Pennell DJ, Rumberger JA, Ryan T, Verani MS; American Heart Association Writing Group on Myocardial Segmentation and Registration for Cardiac Imaging.. Standardized myocardial segmentation and nomenclature for tomographic imaging of the heart. A statement for healthcare professionals from the Cardiac Imaging Committee of the Council on Clinical Cardiology of the American Heart Association. *Circulation*. 2002;105:539-542.
4. O'Gara PT, Kushner FG, Ascheim DD *et al*; American College of Cardiology Foundation/American Heart Association Task Force on Practice Guidelines. 2013 ACCF/AHA guideline for the management of ST-elevation myocardial infarction: a report of the American College of Cardiology Foundation/American Heart Association Task Force on Practice Guidelines. *Circulation*. 2013;127:e362–e425.
5. King SB 3rd, Smith SC Jr, Hirshfeld JW Jr *et al*; 2005 WRITING COMMITTEE MEMBERS, Feldman TE, Kern MJ, O'Neill WW *et al*. 2007 Focused Update of the ACC/AHA/SCAI 2005 Guideline Update for Percutaneous Coronary Intervention: a report of the American College of Cardiology/American Heart Association Task Force on Practice Guidelines: 2007 Writing Group to Review New Evidence and Update the ACC/AHA/SCAI 2005 Guideline Update for Percutaneous Coronary Intervention, Writing on Behalf of the 2005 Writing Committee. *Circulation*. 2008;117:261–295.
6. Messroghli DR, Greiser A, Fröhlich M, Dietz R, Schulz-Menger J. Optimization and validation of a fully-integrated pulse sequence for modified look-locker inversion-recovery (MOLLI) T1 mapping of the heart. *J Magn Reson Imaging*. 2007;26:1081–1086.
7. Messroghli DR, Walters K, Plein S, Sparrow P, Friedrich MG, Ridgway JP, Sivananthan MU. Myocardial T1 mapping: application to patients with acute and chronic myocardial infarction. *Magn Reson Med*. 2007;58:34–40.
8. Xue H, Guehring J, Srinivasan L, Zuehlsdorff S, Saddi K, Chefdhotel C, Hajnal JV, Rueckert D. Evaluation of rigid and non-rigid motion compensation of cardiac perfusion MRI. *Med Image Comput Comput Assist Interv*. 2008;11:35–43.
9. Chefd'hotel C, Hermosillo G, Faugeras O. Flows of diffeomorphisms for multimodal image registration. *Proc IEEE Int Symp Biomed Imaging*. 2002:753-756.
10. Giri S, Chung YC, Merchant A, Mihai G, Rajagopalan S, Raman SV, Simonetti OP. T2 quantification for improved detection of myocardial oedema. *J Cardiovasc Magn Reson*. 2009;11:56.
11. Verhaert D, Thavendiranathan P, Giri S, Mihai G, Rajagopalan S, Simonetti OP, Raman SV. Direct T2 quantification of myocardial oedema in acute ischemic injury. *JACC Cardiovasc Imaging*. 2011;4:269-278.
12. Kellman P, Arai AE, McVeigh ER, Aletras AH. Phase-sensitive inversion recovery for detecting myocardial infarction using gadolinium-delayed hyperenhancement. *Magn Reson Med.* 2002;47:372-383.
13. Flett AS, Hasleton J, Cook C, Hausenloy D, Quarta G, Ariti C, Muthurangu V, Moon JC. Evaluation of techniques for the quantification of myocardial scar of differing etiology using cardiac magnetic resonance. *JACC Cardiovasc Imaging*. 2011;4:150-156.
14. Ugander M, Oki AJ, Hsu L, Kellman P, Greiser A, Aletras AH, Sibley CT, Chen MY, Bandettini WP, Arai AE. Extracellular volume imaging by magnetic resonance imaging provides insights into covert and sub-clinical myocardial pathology. *Eur Heart J*. 2012;33:1268-1278.
15. Wong TC, Piehler K, Meier CG, Testa SM, Klock AM, Aneizi AA, Shakesprere J, Kellman P, Shroff SG, Schwartzman DS, Mulukutla SR, Simon MA, Schelbert EB. Association between extracellular matrix expansion quantified by cardiovascular magnetic resonance and short-term mortality. *Circulation*. 2012;126:1206-1216.
16. Treibel TA, Fontana M, Maestrini V et al. Synthetic ECV - simplifying ECV quantification by deriving haematocrit from T1 blood. In: 10^th^ British Society of Cardiovascular Magnetic Resonance, Annual Meeting, 29 April 2015, London. *Heart.* 2015;101:A16-A17.
17. Payne AR, Casey M, McClure J, McGeoch R, Murphy A, Woodward R, Saul A, Bi X, Zuehlsdorff S, Oldroyd KG, Tzemos N, Berry C. Bright-blood T2-weighted MRI has higher diagnostic accuracy than dark-blood short tau inversion recovery MRI for detection of acute myocardial infarction and for assessment of the ischemic area at risk and myocardial salvage. *Circ Cardiovasc Imaging*. 2011;4:210–219.
18. Dall'Armellina E, Piechnik SK, Ferreira VM, Si QL, Robson MD, Francis JM, Cuculi F, Kharbanda RK, Banning AP, Choudhury RP, Karamitsos TD, Neubauer S. Cardiovascular magnetic resonance by non contrast T1-mapping allows assessment of severity of injury in acute myocardial infarction. *J Cardiovasc Magn Reson*. 2012;14:15.
19. Wassmuth R, Prothmann M, Utz W, Dieringer M, von Knobelsdorff-Brenkenhoff F, Greiser A, Schulz-Menger J. Variability and homogeneity of cardiovascular magnetic resonance myocardial T2-mapping in volunteers compared to patients with edema. *J Cardiovasc Mang Reson*. 2013;15:27.
20. Eitel I, Desch S, Fuernau G, Hildebrand L, Gutberlet M, Schuler G, Thiele H. Prognostic significance and determinants of myocardial salvage assessed by cardiovascular magnetic resonance in acute reperfused myocardial infarction. *J Am Coll Cardiol*. 2010;55:2470-2479.
21. Francone M, Bucciarelli-Ducci C, Carbone I, Canali E, Scardala R, Calabrese FA, Sardella G, Mancone M, Catalano C, Fedele F, Passariello R, Bogaert J, Agati L. Impact of primary coronary angioplasty delay on myocardial salvage, infarct size, and microvascular damage in patients with ST-segment elevation myocardial infarction: insight from cardiovascular magnetic resonance. *J Am Coll Cardiol*. 2009;54:2145-2153.
22. Payne AR, Berry C, Doolin O, McEntegart M, Petrie MC, Lindsay MM, Hood S, Carrick D, Tzemos N, Weale P, McComb C, Foster J, Ford I, Oldroyd KG. Microvascular resistance predicts myocardial salvage and infarct characteristics in ST-elevation myocardial infarction. *J Am Heart Assoc*. 2012;1:e002246.
23. van Kranenburg M, Magro M, Thiele H et al. Prognostic value of microvascular obstruction and infarct size, as measured by CMR in STEMI patients. *JACC Cardiovasc Imaging*. 2014;7:930-939.
24. Medical Research Council. Guidelines for good clinical practice in clinical trials. Available at: <http://www.mrc.ac.uk/documents/pdf/good-clinical-practice-in-clinical-trials/>. Accessed May 2011.

# Supplemental Tables

**Supplemental Table S1.** CMR findings at baseline (n=140) and at 6 months (n=131) in STEMI patients grouped by tertiles of remote zone ECV (%) at baseline.

| Characteristics* |  | STEMI patient tertiles,  remote zone ECV at baseline | | | P-value |
| --- | --- | --- | --- | --- | --- |
|  | All patients | ≤24.2% | >24.2 to ≤26.4% | >26.4% |  |
|  | n=140 | n=46 | n=47 | n=47 |  |
| *CMR findings 2 days post-MI (n=140)* |  |  |  |  |  |
| LV ejection fraction, % | 56±9 | 57±8 | 56±9 | 54±10 | 0.182 |
| LV end-diastolic volume, ml |  |  |  |  |  |
| Men | 162±34 | 158±41 | 162±31 | 167±23 | 0.532 |
| Women | 125±24 | 121±22 | 133±26 | 120±23 | 0.389 |
| LV end-systolic volume, ml |  |  |  |  |  |
| Men | 75±26 | 70±30 | 76±26 | 81±21 | 0.213 |
| Women | 53±16 | 56±14 | 53±16 | 53±18 | 0.955 |
| LV mass, g |  |  |  |  |  |
| Men | 146±37 | 147±42 | 146±31 | 144±36 | 0.753 |
| Women | 101±22 | 107±31 | 105±23 | 97±20 | 0.699 |
| *Edema and infarct characteristics* |  |  |  |  |  |
| Myocardial edema, % LV mass | 31±11 | 29±12 | 30±11 | 35±9 | 0.033 |
| Infarct size, % LV mass | 17±12 | 17±12 | 16±12 | 18±12 | 0.606 |
| Myocardial salvage, % LV mass | 19±8 | 18±7 | 18±8 | 22±9 | 0.025 |
| Myocardial salvage index, % LV mass | 63±23 | 61±20 | 63±22 | 65±25 | 0.585 |
| Late microvascular obstruction present, n (%) | 70 (50) | 26 (57) | 20 (43) | 24 (51) | 0.412 |
| Late microvascular obstruction, % LV mass | 2.3±4.5 | 1.9±4.5 | 2.4±4.7 | 2.5±4.3 | 0.804 |
| *Myocardial native T1 and T2 values* |  |  |  |  |  |
| T1 remote (all subjects, ms) | 961±24 | 953±20 | 963±26 | 967±23 | 0.010 |
| Men | 959±24 | 952±20 | 963±27 | 966±25 | 0.025 |
| Women | 967±21 | 969±17 | 965±25 | 969±20 | 0.866 |
| T1 infarct, ms | 1100±51 | 1103±53 | 1103±51 | 1096±48 | 0.737 |
| T1 hypointense core present, n (%) | 74 (53) | 24 (52) | 23 (49) | 27 (57) | 0.704 |
| T1 hypointense infarct core, ms | 1004±63 | 1001±61 | 1007±68 | 1005±64 | 0.939 |
| T2 remote, ms | 49.9±2.1 | 48.9±1.9 | 50.1±2.3 | 50.7±1.9 | <0.001 |
| *Myocardial ECV values* |  |  |  |  |  |
| ECV remote (all subjects), % | 25.6±2.8 | 22.5±1.3 | 25.4±0.6 | 28.7±1.7 | <0.001 |
| Men | 25.2±1.8 | 22.6±1.3 | 25.4±0.6 | 28.8±1.8 | <0.001 |
| Women | 26.9±2.5 | 22.1±1.1 | 25.4±0.7 | 28.7±1.5 | <0.001 |
| ECV infarct, % | 51.4±8.9 | 50.4±10.1 | 51.0±7.5 | 52.9±9.0 | 0.363 |
| ECV hypointense infarct core, % | 43.2±12.1 | 40.4±9.7 | 44.2±13.5 | 44.9±12.9 | 0.390 |
| ECV infarct zone out-with the infarct core, % | 55.8±11.6 | 53.6±11.5 | 55.7±11.3 | 58.2±11.9 | 0.155 |
| *CMR findings 6 months post-MI (n=131)* |  |  |  |  |  |
| LV ejection fraction at 6 months, % | 63±9 | 63±9 | 63±9 | 63±10 | 0.988 |
| LV end-diastolic volume at 6 months, ml |  |  |  |  |  |
| Men | 165±43 | 164±56 | 164±36 | 168±30 | 0.456 |
| Women | 123±18 | 109±14 | 130±16 | 121±18 | 0.157 |
| LV end-systolic volume at 6 months, ml |  |  |  |  |  |
| Men | 65±36 | 64±46 | 64±30 | 67±25 | 0.376 |
| Women | 43±17 | 40±21 | 46±14 | 42±18 | 0.585 |
| *Myocardial ECV values at 6 months* |  |  |  |  |  |
| ECV remote at 6 months (all subjects), % | 25.7±2.8 | 24.3±2.4 | 25.3±2.3 | 27.6±2.5 | <0.001 |
| Men | 25.4±2.7 | 24.3±2.4 | 25.2±2.4 | 27.1±2.5 | <0.001 |
| Women | 26.9±2.7 | 24.2±1.0 | 25.4±2.3 | 28.3±2.4 | 0.002 |
| ECV infarct at 6 months, % | 51.4±10.3 | 51.9±10.1 | 49.6±10.2 | 52.6±10.7 | 0.368 |

Footnote: Abbreviations: ECV = extracellular volume, LV = left ventricle, T1 = longitudinal relaxation time, T2 = transverse relaxation time. Myocardial edema was measured with T2-mapping.

Data are given as n (%) or mean±SD, as appropriate. *P-values were obtained from one-way ANOVA, Kruskal-Wallis test, or Fischer’s test. 6 month ECV values were available in 131 patients.

**Supplemental Table S2.** Multivariable association of patient characteristics at initial presentation with baseline remote zone ECV assessed by CMR 2 days after reperfusion (n=140).

| Multivariable associations | coefficient (95% CI) | P-value |
| --- | --- | --- |
| *Patient characteristics, angiographic data and LV ejection fraction* | | |
| Male sex | -1.85 (-2.91, -0.79) | 0.001 |
| BMI, kg/m^2^ | -0.12 (-0.22, -0.02) | 0.018 |
| Diabetes mellitus | 1.82 (0.43, 3.20) | 0.010 |
| LV ejection fraction, % | -0.08 (-0.13, -0.03) | 0.002 |
| *Patient characteristics, angiographic data and myocardial edema* | | |
| Male sex | -1.61 (-2.63, -0.60) | 0.002 |
| BMI, kg/m^2^ | -0.14 (-0.24, -0.04) | 0.006 |
| Diabetes mellitus | 1.85 (0.49, 3.21) | 0.008 |
| History of MI | 1.90 (0.19, 3.62) | 0.030 |
| Myocardial edema, % LV mass | 0.09 (0.05, 0.14) | <0.001 |
| *Patient characteristics, angiographic data and remote zone T2* | | |
| Male sex | -1.41 (-2.47, -0.35) | 0.010 |
| Diabetes mellitus | 1.53 (0.14, 2.93) | 0.032 |
| T2 remote, ms | 0.35 (0.14, 0.57) | 0.001 |
| *Patient characteristics, angiographic data and remote zone T1* | | |
| Male sex | -1.22 (-2.28, -0.16) | 0.024 |
| BMI, kg/m^2^ | -0.13 (-0.23, -0.03) | 0.010 |
| Diabetes mellitus | 1.70 (0.32, 3.07) | 0.016 |
| T1 remote, ms | 0.04 (0.02, 0.06) | <0.001 |

Footnote: Abbreviations: BMI = body mass index, CI = confidence interval, LV = left ventricle, MI = myocardial infarction, T1 = longitudinal relaxation time, T2 = transverse relaxation time.

The coefficient (95% confidence intervals) indicates the magnitude and direction of the difference in remote zone ECV(%) for the patient characteristic (binary or continuous).

The clinical characteristics that were univariably associated with remote zone ECV at baseline that were also included in the model were age (p=0.406), current smoker (p=0.483), hypertension (p=0.114), hypercholesterolemia (p=0.348), previous angina (p=0.335), previous MI (p=0.217), previous PCI (p=0.884), symptom onset to reperfusion time (p=0.637), systolic blood pressure at initial angiography (p=0.508), heart rate (p=0.589), TIMI coronary flow grade 2 at initial angiography vs. TIMI coronary flow grade 0/1 at initial angiography (reference category) (p=0.530), TIMI coronary flow grade 3 at initial angiography vs. TIMI coronary flow grade 0/1 at initial angiography (reference category) (p=0.342), TIMI coronary flow grade 2 post-PCI vs. TIMI coronary flow grade 0/1 post-PCI (reference category) (p=0.810), TIMI coronary flow grade 3 post-PCI vs. TIMI coronary flow grade 0/1 post-PCI (reference category) (p=0.777), no ST-segment resolution vs. complete ST-segment resolution (reference category) (p=0.528), incomplete ST-segment resolution vs. complete ST-segment resolution (reference category) (p=0.562), Killip class III vs. Killip class I (reference category) (p=0.738), Killip class IV vs. Killip class I (reference category) (p=0.529).

**Supplemental Table S3.** Multivariable regression analysis of clinical characteristics and ΔECV (n=131).

| Multivariable associations | coefficient (95% CI) | P-value |
| --- | --- | --- |
| *Patient characteristics and angiographic data* | | |
| Baseline remote zone ECV, % | -0.48 (-0.62, -0.34) | <0.001 |
| BMI, kg/m^2^ | -0.14 (-0.23, -0.04) | 0.005 |
| No ST-segment resolution | 1.20 (0.09, 2.31) | 0.034 |
| *Patient characteristics, angiographic data and myocardial edema* | | |
| Baseline remote zone ECV, % | -0.62 (-0.76, -0.48) | <0.001 |
| Age, years | 0.03 (0.00, 0.07) | 0.044 |
| BMI, kg/m^2^ | -0.13 (-0.22, -0.04) | 0.004 |
| Myocardial edema, % of LV mass | 0.08 (0.04, 0.11) | <0.001 |

Footnote: Abbreviations: BMI = body mass index, CI = confidence interval, ECV = extracellular volume, LV = left ventricle.

The coefficient (95% confidence intervals) indicates the magnitude and direction of the difference in ΔECV(%) for the patient characteristic (binary or continuous). Lambda = ΔR1_myocardium_/ΔR1_blood_.

Characteristics also included in the model were age (p=0.275), gender (p=0.482), diabetes mellitus (p=0.184), current smoker (p=0.216), hypertension (p=0.767), hypercholesterolemia (p=0.899), previous angina (p=0.646), previous MI (p=0.754), previous PCI (p=0.310), symptom onset to reperfusion time (p=0.260), systolic blood pressure at initial angiography (p=0.626), heart rate (p=0.296), rescue PCI vs. primary PCI (reference category) (p=0.529), TIMI coronary flow grade 2 at initial angiography vs. TIMI coronary flow grade 0/1 at initial angiography (reference category) (p=0.855), TIMI coronary flow grade 3 at initial angiography vs. TIMI coronary flow grade 0/1 at initial angiography (reference category) (p=0.217), sustained ventricular arrhythmia (p=0.240), TIMI coronary flow grade 2 post-PCI vs. TIMI coronary flow grade 0/1 post-PCI (reference category) (p=0.145), TIMI coronary flow grade 3 post-PCI vs. TIMI coronary flow grade 0/1 post-PCI (reference category) (p=0.344), incomplete ST-segment resolution vs. complete ST-segment resolution (reference category) (p=0.355), Killip class II vs. Killip class ( (reference category) (p=0.520), Killip class III vs. Killip class I (reference category) (p=0.380), Killip class IV vs. Killip class I (reference category) (p=0.789).

# Supplemental Figures

**Supplemental Figure S1.**

Acute STEMI patients assessed for eligibility

(n = 178)

No ECV at 6 months follow-up (n = 9)

- No post-contrast T1 map (n = 1)
- Refused (n = 8)

Analysis

Follow-up

CMR (n = 167)

Enrolment with informed consent

CMR with post-contrast T1 map (n = 140)

CMR 6 months post-MI

(n = 132)

Post-contrast T1 maps 6 months post-MI

(n = 131)

CMR without post-contrast T1 map (n = 27)

- Lack of scanner time

No CMR (n = 11)

- Claustrophobia (n = 8)
- Death (n = 1)
- Contraindication to CMR (n = 2)

CONSORT flow diagram.

**Supplemental Figure S2.**

**
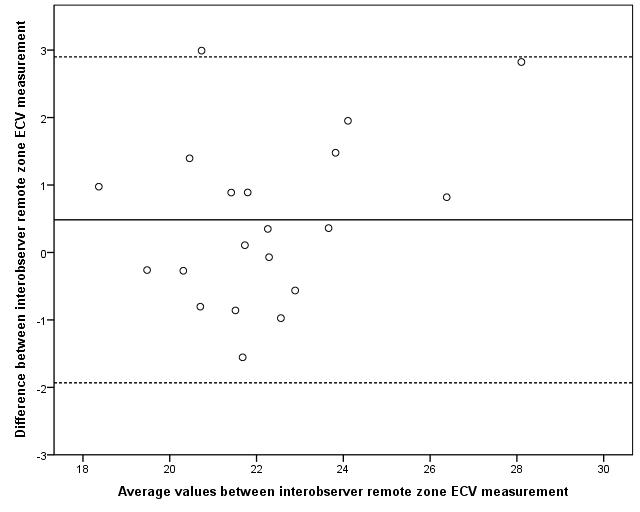
**

Bland-Altman plot for inter-observer variability in remote zone ECV measurement.

**Supplemental Figure S3.**

**
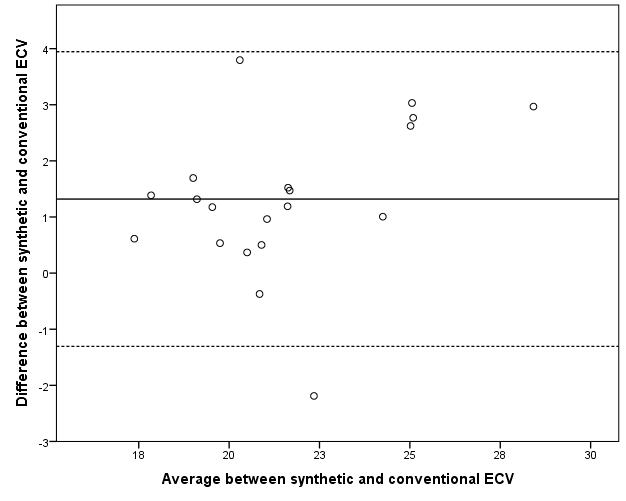
**

Bland-Altman plot for agreement between conventionally measured ECV and synthetic ECV in 20 healthy volunteers.
